# Highlighting Unseen Activity Through 48-Hour Continuous Measurement in Subacute Stroke Rehabilitation: Preliminary Cohort Study

**DOI:** 10.2196/51546

**Published:** 2024-05-29

**Authors:** Emi Mizuno, Takayuki Ogasawara, Masahiko Mukaino, Masumi Yamaguchi, Shingo Tsukada, Shigeru Sonoda, Yohei Otaka

**Affiliations:** 1 Department of Rehabilitation Medicine I School of Medicine Fujita Health University Toyoake Japan; 2 Department of Rehabilitation Medicine II School of Medicine Fujita Health University Toyoake Japan; 3 NTT Basic Research Laboratories and Bio-medical Informatics Research Center NTT Corporation Atsugi Japan; 4 Department of Rehabilitation Medicine Hokkaido University Hospital Sapporo Japan

**Keywords:** activity monitoring, smart clothing system, stroke, unseen, monitoring, recovery, physical condition, deconditioning, muscle wasting, wearable technology, wearable, activity level, rehabilitation, physical therapy, heart rate, ADL, activities of daily living, training, older people, mobile phone

## Abstract

**Background:**

Motor impairments not only lead to a significant reduction in patient activity levels but also trigger a further deterioration in motor function due to deconditioning, which is an issue that is particularly pronounced during hospitalization. This deconditioning can be countered by sustaining appropriate activity levels. Activities that occur outside of scheduled programs, often overlooked, are critical in this context. Wearable technology, such as smart clothing, provides a means to monitor these activities.

**Objective:**

This study aimed to observe activity levels in patients who had strokes during the subacute phase, focusing on both scheduled training sessions and other nontraining times in an inpatient rehabilitation environment. A smart clothing system is used to simultaneously measure heart rate and acceleration, offering insights into both the amount and intensity of the physical activity.

**Methods:**

In this preliminary cohort study, 11 individuals undergoing subacute stroke rehabilitation were enrolled. The 48-hour continuous measurement system, deployed at admission and reassessed 4 weeks later, monitored accelerometry data for physical activity (quantified with a moving SD of acceleration [MSDA]) and heart rate for intensity (quantified with percent heart rate reserve). The measurements were performed using a wearable activity monitoring system, the hitoe (NTT Corporation and Toray Industries, Inc) system comprising a measuring garment (wear or strap) with integrated electrodes, a data transmitter, and a smartphone. The Functional Independence Measure was used to assess the patients’ daily activity levels. This study explored factors such as differences in activity during training and nontraining periods, correlations with activities of daily living (ADLs) and age, and changes observed after 4 weeks.

**Results:**

A significant increase was found in the daily total MSDA after the 4-week program, with the average percent heart rate reserve remaining consistent. Physical activity during training positively correlated with ADL levels both at admission (*ρ*=0.86, *P*<.001) and 4 weeks post admission (*ρ*=0.96, *P*<.001), whereas the correlation between age and MSDA was not significant during training periods at admission (*ρ*=–0.41, *P*=.21) or 4 weeks post admission (*ρ*=–0.25, *P*=.45). Conversely, nontraining activity showed a negative correlation with age, with significant negative correlations with age at admission (*ρ*=–0.82, *P*=.002) and 4 weeks post admission (*ρ*=–0.73, *P*=.01).

**Conclusions:**

Inpatient rehabilitation activity levels were positively correlated with ADL levels. Further analysis revealed a strong positive correlation between scheduled training activities and ADL levels, whereas nontraining activities showed no such correlation. Instead, a negative correlation between nontraining activities and age was observed. These observations suggest the importance of providing activity opportunities for older patients, while it may also suggest the need for adjusting the activity amount to accommodate the potentially limited fitness levels of this demographic. Future studies with larger patient groups are warranted to validate and further elucidate these findings.

## Introduction

Motor impairments, such as those following a stroke, often lead to a significant reduction in patient activity levels. This reduced activity often triggers a further deterioration in motor function due to deconditioning—an issue that is particularly pronounced during hospitalization [1-3]. Various factors contribute to this decline in activity during inpatient care, including decreased activity capacity due to motor impairment, a limited range of available activities because of treatment constraints, and the constant presence of caregivers, allowing patients to become overly reliant.

Thus, maintaining and enhancing activity levels is crucial to managing the challenges of deconditioning during hospitalization. Within this context, inpatient rehabilitation has a vital role in enabling skill acquisition, and promoting and elevating activity levels by offering opportunities for activity. Given the profound impact of activity volume on both motor and cognitive functions [4-6], activities outside of structured rehabilitation sessions must also be considered as part of the rehabilitation process. In reality, activities outside of structured sessions have not received sufficient attention despite their importance. For instance, a study on inpatients with spinal cord injury reported a tendency for individuals to remain sedentary outside structured rehabilitation sessions [7]. Additionally, qualitative research reveals that while stroke survivors acknowledge the necessity of rest during inpatient rehabilitation, many of them experience excessive downtime, which contributes to boredom and frustration [8]. Therefore, increasing the amount of activity by encouraging more active engagement during free time may contribute to enhancing the overall functional outcomes of inpatient rehabilitation. According to a qualitative study, factors identified as influencing participation in these activities include patients’ impairments, attitudes toward activities, personal preferences, and their environment [9]. Despite the recognition of these factors, more objective research is critically needed to understand the determinants of patients’ activity levels and optimize strategies to maximize the activities.

Fortunately, recent technological advancements have made activity measurement more attainable. Heart rate and accelerometry measurements are the 2 primary methods used to evaluate activity. Changes in heart rate, reflecting fluctuations in cardiac input, indicate activity intensity. Wearable devices such as chest monitors and wristband-type plethysmographs have been widely used for heart rate-based activity monitoring [10,11]. On the other hand, accelerometers offer insight into the quantity of physical activity, demonstrating high levels of reliability and validity [12,13]. The number of steps taken is commonly used as an activity metric using acceleration. However, for patients who had strokes with slow walking speeds and irregular gait patterns, this measure may not be entirely accurate [14]. As a result, alternative metrics like “activity count,” moving SD of acceleration (MSDA), or root mean square might be more appropriate for quantifying activity in certain contexts [15-17]. Prior studies have shown that heart rate and acceleration-measured activity values can behave differently [18], and that these indices can complement each other in estimating energy expenditure [19,20]. Thus, simultaneous measurement of these indices may offer valuable insights into the activity patterns of patients who had strokes.

This study set out to monitor activity over the entirety of the day, inclusive of both training and nontraining periods, in subacute rehabilitation inpatients. We aimed to evaluate the fluctuations in activity patterns before and after a pre-established period of inpatient rehabilitation. To accomplish this, we used the smart clothing system (hitoe system), capable of concurrently measuring heart rate and acceleration over prolonged durations.

## Methods

### Participants

This study involved 11 patients who had strokes, excluding those with subarachnoid hemorrhage, who were admitted to the comprehensive inpatient rehabilitation ward of Fujita Medical University Nanakuri Memorial Hospital between March 2, 2018, and November 17, 2020. The inclusion criteria required participants to have entered the comprehensive inpatient rehabilitation ward within 60 days of stroke onset and consented to participate in this study. The patient demographic comprised 5 males and 6 females (Table 1). Among these, 9 and 2 patients had cerebral infarction and hemorrhage, respectively. The participants’ mean age was 64.5 (SD 12.0) years. Upon ward admission, the patients exhibited a mean Functional Independence Measure (FIM) total score of 52.2 (SD 16.8).

**Table 1:** Demographic variables.

Characteristics		Values
Age (years), mean (SD)		65 (13)
**Sex, n**		
	Male	5
	Female	6
**Diseases, n**		
	Ischemic stroke	9
	Hemorrhagic stroke	2
SIAS^a^, mean (SD)		13.6 (6.1)
**FIM^b^ total score, mean (SD)**		79.6 (19.3)
	Motor	52.2 (16.6)
	Cognitive	27.5 (7.0)

^a^SIAS: Stroke Impairment Assessment Set.

^b^FIM: Functional Independence Measure.

### Measurement

**Figure 1:**
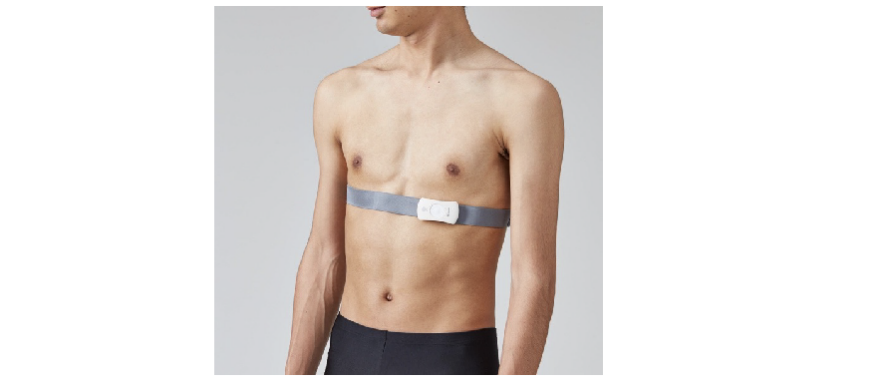
Measurement setting: the system is composed of wearable clothing with an embedded electrode and a transmitter that sends the data to a smartphone via Bluetooth.

The measurements were performed using a wearable activity monitoring system, the hitoe system [21,22], which comprises a measuring garment (wear or strap) with integrated electrodes, a hitoe transmitter for data transfer, and a smartphone app (Figure 1). This system measures heart rate based on electrocardiogram waveforms obtained from the built-in electrodes. A chest strap was used as the measuring device in this study. The transmitter has a built-in accelerometer that quantifies activity based on acceleration and posture determination (supine or otherwise) at a sampling rate of 25 Hz. The MSDA of the measured acceleration over a 2-second duration was used to indicate the amount of physical body movement [17].

The activity was measured within 1 week of admission to the convalescent rehabilitation hospital and repeated 4 weeks post admission. Patients wore the hitoe system continuously for 48 hours, excluding bathing times when measurements were halted and resumed postbath. On the second day of measurement, the belt and transmitter were replaced.

The FIM was used to assess the patients’ daily activity levels. Specifically, it assesses the degree of independence of activities on a total of 18 items, including 13 motor and 5 cognitive, on a 7-point scale [23]. This study used the 13 motor items as an index of daily activity level. The training (the time during the rehabilitative training session) and nontraining (the time other than the training period) periods were recorded by the physical and occupational therapists who supervised the patients.

### Analysis

Our study used 48 hours of data measured with the abovementioned system. The mean heart rate was calculated from the measured data for supine, sitting, and standing positions. Additionally, the heart rate was used to quantify exercise intensity, which is a common practice in exercise prescription [24]. The American College of Sports Medicine guidelines recommend using a percent heart rate reserve (%HRR) for measuring exercise intensity using heart rate [25]. The %HRR was calculated as heart rate—resting heart rate or maximum heart rate—resting heart rate, where the maximum heart rate was measured or estimated using age. The resting heart rate was determined using the mean heart rate during the night while lying down (median heart rate when the patient was judged to be lying down between 12 and 5 AM) when measurements were stable. The mean of the 24-hour %HRR values calculated with these values was used to analyze exercise intensity.

### Statistics Analysis

The Wilcoxon signed-rank test was used to test for significant differences, and statistical significance was set at *P*<.05. All statistical analyses were performed using the JMP11 (SAS Institute Inc).

### Ethical Considerations

The Ethics Committee of Fujita Health University (HM17-220) approved this study protocol, ensuring adherence to ethical standards. All participants provided informed consent. Identifiable data were securely stored in a locked cabinet, separate from the analysis data sets that were anonymized, to safeguard privacy and confidentiality. Participants did not receive financial compensation for their involvement in this study.

## Results

**Figure 2:**
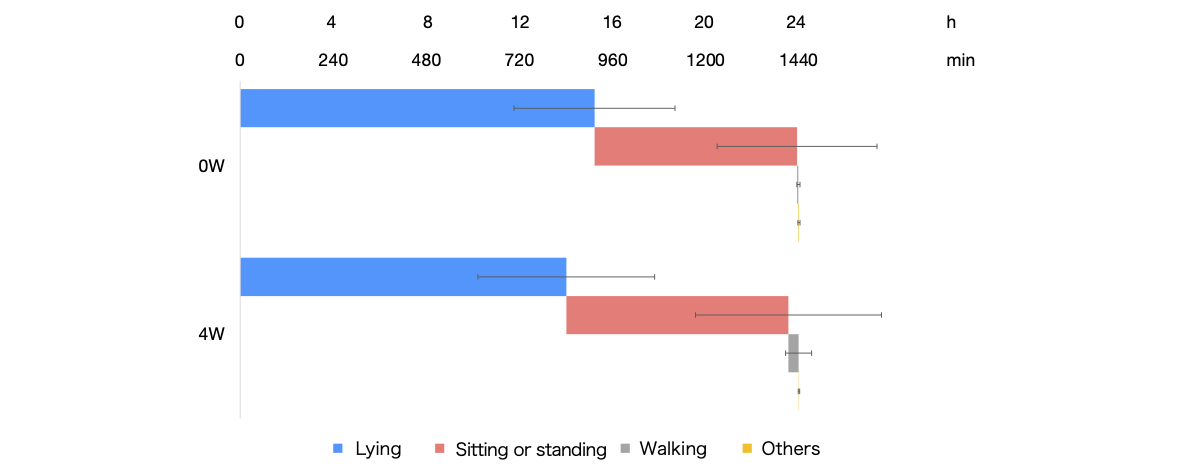
Comparison of the time spent in a supine position, sitting or standing, and walking among inpatients with stroke at admission versus 4 weeks post admission. 0W: at admission; 4W: at 4 weeks.

At admission to the comprehensive inpatient rehabilitation ward, the daily average of lying, sitting or standing, and walking times were 913.1 (SD 207.5) minutes, 522.0 (SD 206.0) minutes, and 3.5 (SD 4.0) minutes, respectively, and were 840.3 (SD 227.7) minutes, 572.8 (SD 239.7) minutes, and 26.0 (SD 33.5) minutes, respectively, at 4 weeks post admission (*P*=.30, *P*=.49, and *P*=.05: Figure 2).

**Figure 3:**
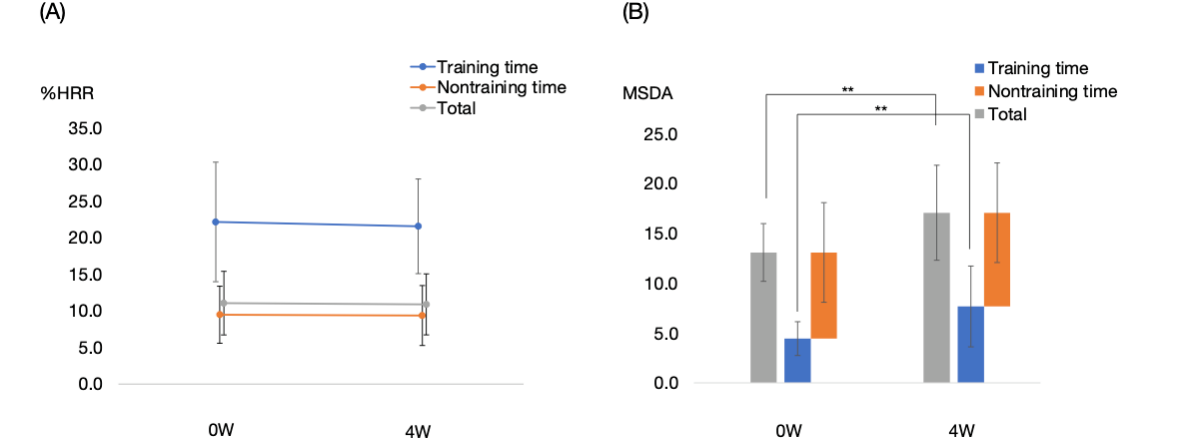
Graphs of the mean exercise intensity and physical activity of the inpatients who had stroke at admission versus 4 weeks post admission. (A) Averaged %HRR at admission and 4 weeks post admission. (B) Daily total MSDA at admission and 4 weeks post admission. %HRR: percent heart rate reserve; 0W: at admission; 4W: at 4 weeks; MSDA: moving SD of acceleration.

The mean %HRR indicating exercise intensity at admission and 4 weeks post admission were 11.05% (SD 4.34%) and 10.88% (SD 4.16%), respectively, and did not change significantly (*P*=.83: Figure 3). Furthermore, the mean %HRR during rehabilitation training and nontraining periods did not change significantly at admission compared with 4 weeks post admission (training period: 22.13%, SD 8.16%, and 21.54%, SD 6.46%, *P*=.90; nontraining period: 9.47%, SD 3.89%, and 9.36%, SD 4.09%, *P*=.83). However, the MSDA daily total and during training increased significantly (daily total: from 13.09, SD 2.90, at admission to 17.08, SD 4.77, at 4 weeks post admission, *P*=.003; during training: from 4.44, SD 1.70, at admission to 7.67, SD 4.06, at 4 weeks post admission, *P*=.005), while that during the nontraining period did not change significantly (8.65, SD 1.84, at admission and 9.41, SD 2.96, at 4 weeks post admission, *P*=.47).

**Figure 4:**
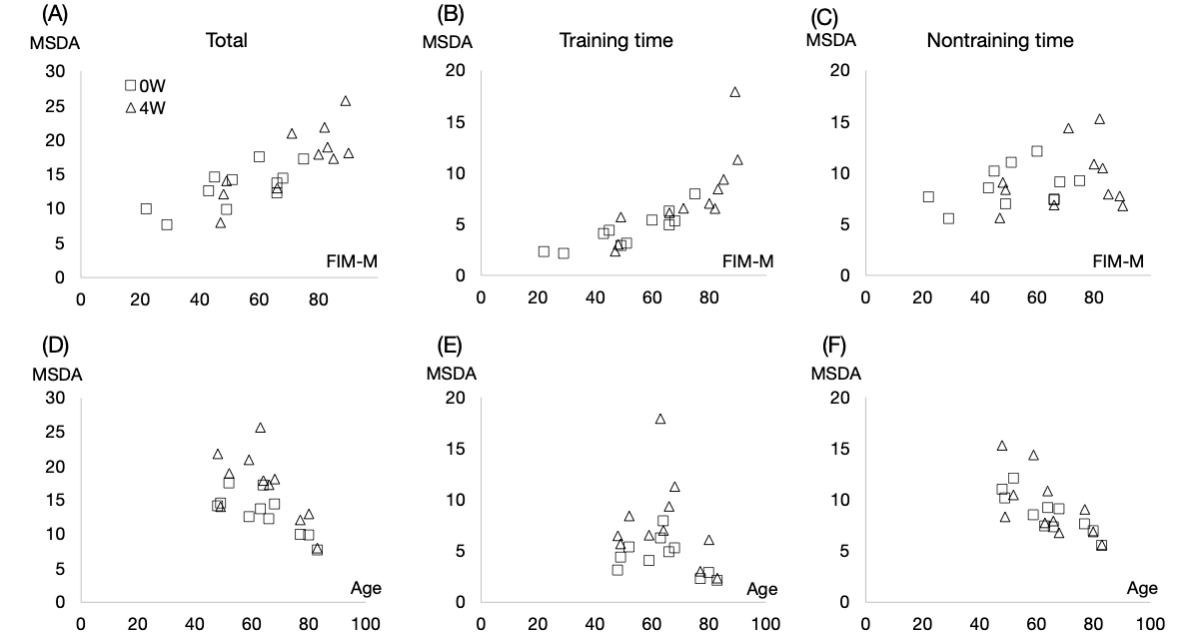
Correlations between the MSDA and FIM-M and age of the inpatients who had stroke at admission (square) and 4 weeks post admission (triangle). (A)-(C) correlation between MSDA and FIM-M in daily total (A), during training (B), and nontraining periods (C). (D)-(F) correlation between MSDA and FIM-M in daily total (D) and during the training (E) and nontraining (F) periods. 0W: at admission; FIM-M: Functional Independence Measure motor score; MSDA: moving SD of acceleration.

The MSDA during training strongly correlated with FIM both at admission (*ρ*=0.86, *P*<.001) and 4 weeks post admission (*ρ*=0.96, *P*<.001; Table 2, Figure 4). Similarly, a correlation was observed between the MSDA and FIM at 4 weeks post admission (*ρ*=0.73, *P*=.01), while the correlation was marginal at admission (*ρ*=0.58, *P*=.06).

On the other hand, a significant negative correlation was noted between age and MSDA both at admission (*ρ*=–0.71, *P*=.02) and 4 weeks post admission (*ρ*=–0.70, *P*=.02). In the context of nontraining activities, the correlation was even stronger, with significant negative correlations with age at admission (*ρ*=–0.82, *P*=.002) and 4 weeks post admission (*ρ*=–0.73, *P*=.01). Conversely, the correlation between age and MSDA was not significant during training periods at admission (*ρ*=–0.41, *P*=.21) or 4 weeks post admission (*ρ*=–0.25, *P*=.45).

**Table 2:** Spearman rank correlation coefficients between MSDA^a^ versus FIM^b^ and age at admission and 4 weeks post admission.

	Versus FIM				Versus age			
MSDA	0W^c^		4W^d^		0W		4W	
	*ρ^e^*	*P* value	*ρ*	*P* value	*ρ*	*P* value	*ρ*	*P* value
Total	0.58	.06	0.73^f^	.01	–0.71^f^	.02	–0.70^f^	.02
Training	0.86^g^	<.001	0.96^g^	<.001	–0.41	.21	–0.25	.45
Nontraining	0.27	.42	0.01	.98	–0.82^g^	.002	–0.73^f^	.01

^a^MSDA: moving standard deviation of acceleration.

^b^FIM: Functional Independence Measure.

^c^0W: at admission.

^d^4W: at 4 weeks.

^e^*ρ*: Spearman rank correlation coefficient.

^f^*P*<.05.

^g^*P*<.01.

## Discussion

### Principal Findings

This study used the hitoe system to continuously monitor the heart rate and activity status of 11 patients who had strokes and who were admitted to a comprehensive inpatient rehabilitation ward for over 48 hours at admission and 4 weeks post admission. A significant increase was observed in activity time and total physical activity at 4 weeks post admission compared to that upon admission. However, no significant change in the average physical activity intensity was observed. Additionally, the increase in total physical activity primarily resulted from increased activity during training. A significant correlation was found between activity during the training period and the FIM, but not for activity during the nontraining period. Instead, activity during the nontraining period negatively correlated with age.

The activity levels of patients who had strokes, along with their long-term changes, have been thoroughly examined using accelerometers. It is well established that activity levels of patients with stroke are generally lower than those of healthy individuals [1,2]. These levels typically decrease during the acute phase and rise slightly over the following 3 months, but do not significantly increase afterward, remaining lower than in healthy individuals [26]. Variations have been observed in the postdischarge activity levels following acute treatment. In some cases, activity levels increase over time, while in less active groups, there is a further decrease in activity after discharge [3,27].

Heart rate, as a reflection of exercise intensity, signifies changes in cardiac output, and thus, represents the effort required for exercise in comparison to the absolute activity measured by acceleration [28]. As such, heart rate can offer valuable insights into a stroke patient’s mobility function since it quantifies the effort exerted in movement. For example, patients who had strokes with mild motor impairment may move relatively efficiently, whereas those with severe impairment may struggle to move despite expending a lot of effort.

In this study, we noticed an upward trend in physical activity, as measured by an accelerometer, from admission to 4 weeks later. Nevertheless, a significant increase was only observable during training sessions. The strong correlation between body movement measured by an accelerometer during training and the FIM could indicate a rise in dynamic body movement associated with the ability to perform activities of daily living (ADL). Patients who had strokes usually experienced incremental improvements in basic motor skills [29-31], mirrored by an increased walking speed and frequency of standing up, contributing to improved accelerometer readings [32,33]. Additionally, enhancements in exercise tolerance could lead to better accelerometer measurements since individuals can move for longer durations with fewer breaks [34,35].

Interestingly, the amount of activity during the nontraining time did not correlate with FIM but displayed a strong negative correlation with age, and several factors may be attributable to this age-related decrease in activity. Older individuals are more likely to experience conditions, including sarcopenia [36-38] and osteoarthritis [39,40], that could limit their basic fitness and reduce voluntary exercise. Furthermore, they may also actively avoid activities that require significant effort and cause fatigue [41,42]. From these insights, 2 key implications emerge. First, for older patients who can manage, engaging in additional activities during nontraining periods could be beneficial to boost their exercise tolerance, given the importance of maintaining activity levels for improving motor function and exercise tolerance [43,44]. Second, the data also suggests that the daily energy output of older adults may be naturally restricted. Considering this, some individuals might find it beneficial to manage their daily activities based on an “Energy Conservation Strategy.” This approach, commonly used for patients grappling with fatigue issues, focuses on the strategic allocation of energy throughout the day [45].

Despite the increase in physical activity, the average exercise intensity remained relatively unchanged, which could be due to improved motor efficiency following stroke and rehabilitation, enabling patients to perform daily activities more efficiently. Thus, the measurement of both acceleration and heart rate may be used to monitor changes in efficiency, which is observed through the process of poststroke rehabilitation [46,47]. The lack of change in heart rate might also result from reduced effort in certain segments of the population, potentially due to factors such as depression, low motivation, and pain—issues frequently associated with poststroke conditions [48]. Further investigation, such as comparisons with age-matched healthy populations, may help explore the factors that kept the exercise intensity unchanged.

### Limitations

This study had some limitations. First, a small sample size was used in this study, which precluded subgroup analysis. Consequently, biases such as the severity of illness may have influenced the results. For instance, previous studies have reported that acceleration-measured exercise intensity did not correlate with heart rate at low exercise intensities rather than at high exercise intensities, complicating the generalization of these findings to all patients who had strokes [26]. Therefore, the current analysis should be considered preliminary, and a more comprehensive investigation into the factors influencing activity increase needs to be conducted with larger sample sizes in the future. Nevertheless, the findings of this study still have important implications, emphasizing the importance of simultaneous measurement of heart rate and acceleration, which may reflect the efficiency of daily activities, as well as the need to monitor patients’ nontraining activities during rehabilitation programs.

### Conclusions

Rehabilitation inpatients experienced a noticeable increase in activity volume during the subacute poststroke phase, even while exercise intensity remained fairly steady. The rise in activity that corresponds with ADL was primarily driven by scheduled training. Activity volume during nontraining periods did not correlate with ADLs; however, a strong correlation was noted with age, highlighting the significance of providing opportunities for activity, particularly for older patients. These observations may also suggest the need for adjusting the activity amount to accommodate the potentially limited fitness levels of this demographic. Future studies with larger and more varied patient groups are warranted to validate and further elucidate these findings.

## Abbreviations

%HRR: percent heart rate reserve
ADL: activities of daily living
FIM: Functional Independence Measure
MSDA: moving standard deviation of acceleration

## Data Availability

The data that support the findings of this study are available from the corresponding author upon reasonable request.
